# Luminescence and X-ray Absorption Properties of Uniform Eu^3+^:(H_3_O)Lu_3_F_10_ Nanoprobes

**DOI:** 10.3390/nano9081153

**Published:** 2019-08-12

**Authors:** Daniel González-Mancebo, Ana Isabel Becerro, Ariadna Corral, Marcin Balcerzyk, Manuel Ocaña

**Affiliations:** 1 Instituto de Ciencia de Materiales de Sevilla (CSIC-US), c/Américo Vespucio, 49. 41092 Sevilla, Spain; 2 Centro Nacional de Aceleradores (CNA) (Universidad de Sevilla, Junta de Andalucía, CSIC), c/Thomas Alva Edison 7, 41092 Sevilla, Spain

**Keywords:** nanoparticles, rare earth fluorides, lutetium, polyols, luminescence, X-ray computed tomography

## Abstract

Due to the high atomic number of lutetium and the low phonon energy of the fluoride matrix, Lu-based fluoride nanoparticles doped with active lanthanide ions are potential candidates as bioprobes in both X-ray computed tomography and luminescent imaging. This paper shows a method for the fabrication of uniform, water-dispersible Eu^3+^:(H_3_O)Lu_3_F_10_ nanoparticles doped with different Eu contents. Their luminescent properties were studied by means of excitation and emission spectra as well as decay curves. The X-ray attenuation capacity of the phosphor showing the highest emission intensity was subsequently analyzed and compared with a commercial contrast agent. The results indicated that the 10% Eu^3+^-doped (H_3_O)Lu_3_F_10_ nanoparticles fabricated with the proposed polyol-based method are good candidates to be used as dual probes for luminescent imaging and X-ray computed tomography.

## 1. Introduction

Due to their unique luminescent properties such as photostability, long fluorescence lifetimes, narrow emission lines, and large Stokes shifts, RE-based (RE = rare-earths, including Y, Sc, and Lanthanides) inorganic nanoparticles are considered very promising candidates for different applications in biomedicine such as bioimaging [1], biosensing [2], and therapy [3]. In particular, RE fluoride nanoparticles have been paid much attention in the last decade because the fluoride matrix exhibits high refractive index and low phonon energy [4]. These properties minimize the probability of non-radiative processes and consequently increase the luminescence quantum yield when compared with other inorganic matrices such as RE oxides [5], hydroxides [6], and phosphates [7].

Ideal nanoparticles to be used in biomedicine should comply with a number of requirements such as nanometer size, uniform size and shape, colloidal stability in physiological fluids, and lack of cytotoxicity, among others [8]. Dramatic efforts have been therefore devoted to the exploration of convenient synthesis methods that render RE fluoride-based nanoparticles with the required features [9]. Among the fluoride matrices, Ln:REF_3_ and Ln:AREF_4_ (Ln: optically active lanthanide, A = Na and K) have been extensively investigated [10,11,12] and a number of synthetic routes have been described, which render adequate nanoparticles to be used in biomedicine. These routes include hydrothermal [13,14] and solvothermal [15,16] synthesis at temperatures above 100 °C. Compared with the successful synthesis and application of Ln:REF_3_ and Ln:AREF_4_ nanoparticles, little attention has been paid to the fabrication of RE fluoride nanoparticles with different stoichiometry such as Ln:ARE_3_F_10_ (A = alkaline and H_3_O) [17,18,19]. Among them, those with RE = Lu would be interesting not only as luminescent bioprobes but also as X-ray computed tomography contrast agents (CT CAs). CAs are sometimes needed during a CT exploration to increase the contrast between the region of interest and its surrounding. Currently used CT CAs are barium salts and iodine complexes that, due to the high atomic number of Ba (Z = 56) and I (Z = 53), offer a high X-ray attenuation capacity. CT CAs based on lutetium compounds, as is the case of ALu_3_F_10_ nanoparticles, are expected to show a high capacity to absorb X-rays due to the high atomic number of Lu (Z = 71, the highest among the RE elements). Two studies have been published on the synthesis and luminescent properties of nanoparticles based on the potassium member (Ln:KLu_3_F_10_) [20,21]. The (H_3_O)Lu_3_F_10_ compound, however, has only been fabricated as a polycrystalline powder using a chimie douce method consisting of the addition of a hydrated lutetium oxalate to a heated solution of HF at any temperature within the 30–100 °C interval [22,23]. To the best of our knowledge, the (H_3_O)Lu_3_F_10_ matrix has not been fabricated as a colloidal nanomaterial, nor has it been evaluated as a potential CT CA or doped with lanthanides to prove its luminescent properties. 

Herein, a simple synthesis method for the fabrication of uniform, water-dispersible (H_3_O)Lu_3_F_10_ nanoparticles is reported. The method consists of the treatment, at just 50 °C for 15 h, of an ethylene glycol/water solution (90/10 v/v) containing lutetium nitrate (0.2 M) and a fluorine precursor (NaBF_4_, 0.18 M) that slowly releases fluorine to the solution. The effect of the ethylene glycol/water ratio, lutetium nitrate concentration, and reaction temperature on the morphology and crystalline structure of the obtained precipitates was evaluated. The same method is shown to be useful for the synthesis of uniform Eu^3+^-doped (H_3_O)Lu_3_F_10_ nanoparticles with similar size and shape. The luminescence and X-ray attenuation properties of such doped nanophosphors were analyzed to assess their suitability for biomedical applications as luminescence bioprobes and as CAs for X-ray computed tomography.

## 2. Materials and Methods 

### 2.1. Materials

Lutetium nitrate hexahydrate (Lu(NO_3_)_3_·6H_2_O, Sigma Aldrich (Sigma-Aldrich Chemie Gmbh, Munich, Germany), purity 99%, Ref. 436429), europium nitrate hydrate (Eu(NO_3_)_3_·*x*H_2_O, Sigma Aldrich, purity 99.9%, Ref. 254061), sodium tetrafluoroborate (NaBF_4_, Sigma Aldrich, purity 98%, Ref. 8.43877), and ethylene glycol (EG) (anhydrous, Sigma Aldrich, purity 99.8%, Ref. 324558) were used as received.

### 2.2. Synthesis of the Nanoparticles

(H_3_O)Lu_3_F_10_ nanoparticles were obtained by homogeneous precipitation in a mixture of EG and water. In a typical procedure, a specific amount of Lu(NO_3_)_3_·6H_2_O was dissolved in 5.4 mL of EG while NaBF_4_ was dissolved in 0.6 mL of water, both under stirring at room temperature to favor dissolution. Both solutions were admixed together (final [Lu(NO_3_)_3_] = 0.2 M and [NaBF_4_] = 0.18 M) in tightly closed test tubes and aged in a conventional oven preheated at 50 °C for 15 h. The resulting dispersion was cooled down to room temperature and the supernatant was removed by centrifugation. The precipitate was then washed twice with ethanol and once with distilled water. Finally, the clean precipitate was redispersed in Milli-Q water or dried at room temperature for certain analyses. The Lu(NO_3_)_3_·6H_2_O concentration as well as the EG/H_2_O ratio and the reaction temperature were varied to analyze their effect on the morphological and structural characteristics of the precipitated nanoparticles.

Eu^3+^-doped (H_3_O)Lu_3_F_10_ nanoparticles were synthesized using the same method but adding the corresponding amounts of Eu(NO_3_)_3_·6H_2_O to the EG solution containing the lutetium salt. Total cation concentration in these solutions was kept constant at 0.2 M while the Eu/(Eu + Lu) molar ratio was varied from 2.5% to 10% to evaluate the effect of the dopant content on the luminescent properties of the nanoparticles.

### 2.3. Characterization Techniques

Transmission electron microscopy (TEM; Philips 200CM) was used to analyze nanoparticle shape as well as to calculate particle-size distributions by measuring the diameter of about one hundred particles. Dynamic light-scattering (DLS) measurements, conducted with a Malvern Zetasizer Nano-ZS90 apparatus in aqueous suspensions of the nanoparticles at pH = 6, were used to calculate the mean hydrodynamic diameter.

The crystalline structure of the prepared particles was identified by X-ray diffraction (XRD) using a PANalytical X’Pert Pro with an X-Celerator detector. The unit cell parameters were determined from the XRD data (collected at intervals of 0.02° (2θ)) by Rietveld refinement. Refined parameters were: Zero of the diffractometer, scale factor, background coefficients, cubic unit cell parameter, and profile parameters. The crystallite size was estimated from the 511 reflection (located at 30.7° 2θ) using the Scherrer formula and LaB_6_ as the external standard.

Excitation and emission spectra were recorded, using a Horiba Jobin Yvon spectrofluorometer (Fluorolog FL3-11), in aqueous suspensions of the Eu^3+^-doped nanoparticles having the same concentration (4 mg·cm^−3^), so that their intensities could be compared to one another. The excitation and emission slits were opened at 1 and 5 nm for the excitation spectra and vice versa for the emission ones. The spectra were corrected by both the spectral response of the detector and the lamp. Luminescence decay curves, recorded with the same equipment but using a pulsed lamp, were registered in powdered samples at the characteristic emission wavelength of Eu^3+^ ions (611 nm) using an excitation wavelength of 394 nm with excitation and emission slits opened at 5 and 1 nm, respectively.

Evaluation of the X-ray absorption capacity of the nanoparticles was carried out in aqueous dispersions containing different concentrations of nanoparticles placed in a multiwell microplate. A well containing Milli-Q water was also measured for calibration. CT images were acquired with a NanoSPECT/CT^®^ (Bioscan) using the following acquisition parameters: 106 mA current, 75 kV voltage, exposure time per projection of 1500 ms, 360 projections per rotation, 6 cm image length, and a pitch of 1. The total acquisition time required was 18 min. The image was reconstructed with VivoQuant image processing software (Invicro), with the exact cone beam filtered back projection algorithm and the Shepp–Logan 98% filter. The resulting image pixel size was uniform in three dimensions at 0.2 mm. Images were analyzed with PMOD 3.9 software (PMOD Technologies LLC). Spherical volumes of interest (VOIs) of 2 mm radius were made within each sample to calculate the X-ray attenuation (in Hounsfield units = HU) for each suspension concentration. Average values of Milli-Q water and nanoparticle suspensions were used to calculate HU values in the images, with attenuation being 0 HU for water and −1000 HU for air.

## 3. Results and Discussion

### 3.1. Synthesis, Morphology, and Crystal Structure of Undoped and Eu^3+^-Doped Lutetium Fluoride Nanoparticles

**Figure 1:**
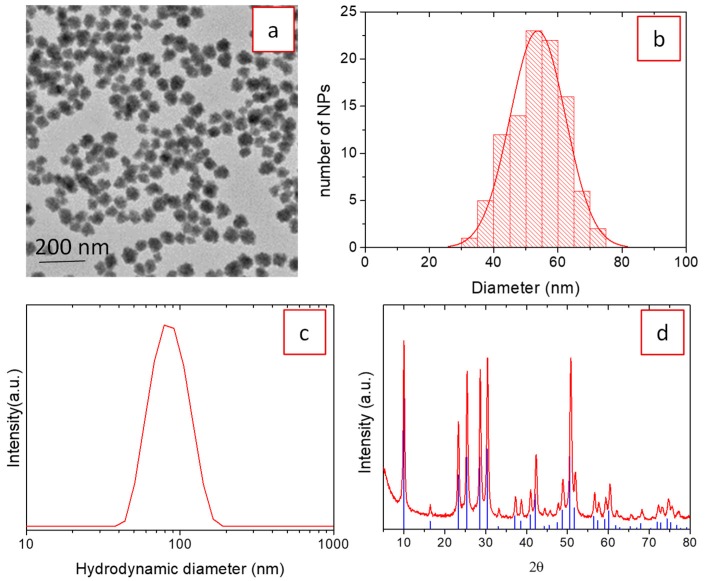
TEM micrograph (**a**), particle size distribution from TEM (**b**), dynamic light-scattering (DLS) curve (**c**), and XRD pattern (**d**) of nanoparticles obtained after treating, at 50 °C for 15 h, an EG/H_2_O (90/10) solution containing Lu(NO_3_)_3_·6H_2_O (0.2 M) and NaBF_4_ (0.18 M). Ticks at the bottom of (d) correspond to cubic (H_3_O)Lu_3_F_10_ (PDF 04-016-7065).

Synthesis of uniform nanoparticles was accomplished here using the homogeneous precipitation method in EG medium. EG is known to act not only as a solvent but also as a capping agent, which helps to limit particle growth [24]. Homogeneous precipitation can be achieved by a controlled release of either anions or cations to the solution in order to get similar precipitation kinetics throughout the reaction medium [25]. We have selected the first approach and used NaBF_4_ as a fluorine source, which slowly releases fluorine anions through hydrolysis of [BF_4_]^−^ [26]. The presence of water in the reaction medium is, therefore, required for the hydrolysis to take place. Homogeneous precipitation requires, in addition, a specific reaction kinetics that must be found by adjusting, through trial and error method, the experimental conditions of the reaction such as solvent nature, temperature, and reagent concentration. Following this methodology and for simplification, we first looked for the experimental parameters necessary to get uniform un-doped (H_3_O)Lu_3_F_10_ nanoparticles. We found that treating an EG solution containing Lu(NO_3_)_3_·6H_2_O (0.2 M), NaBF_4_ (0.18 M), and 10% H_2_O (v/v) at 50 °C for 15 h rendered uniform, spherical nanoparticles, as shown in the TEM micrograph of Figure 1a. The histogram in Figure 1b, obtained by measuring the diameter of more than a hundred particles in TEM images, rendered an average diameter of 55 nm as well as a narrow size distribution (standard deviation ~15 nm), which confirms the uniformity and nanometer character of the precipitated particles. The hydrodynamic diameter obtained from the DLS curve, recorded on an aqueous suspension of the nanoparticles at pH = 6 (Figure 1c), was 80 nm. This value, slightly higher than the average diameter provided by the TEM histogram, indicates a slight aggregation of the nanoparticles in water, although the size of the aggregates is still well below 100 nm. The XRD pattern of the dried precipitate (Figure 1d) indicated that the nanoparticles crystallized as cubic (H_3_O)Lu_3_F_10_. The cubic (H_3_O)Lu_3_F_10_ phase was described by Le Berre [22] and shows a peculiar zeolitic structure, with cavities linked together by tunnels where water molecules are located. The crystallite size, estimated from the width of the 511 reflection located at 30.7° 2θ using the Scherrer equation, was 35 nm. This value, slightly lower than the nanoparticles diameter, indicates that the nanoparticles may be polycrystalline in character.

**Figure 2:**
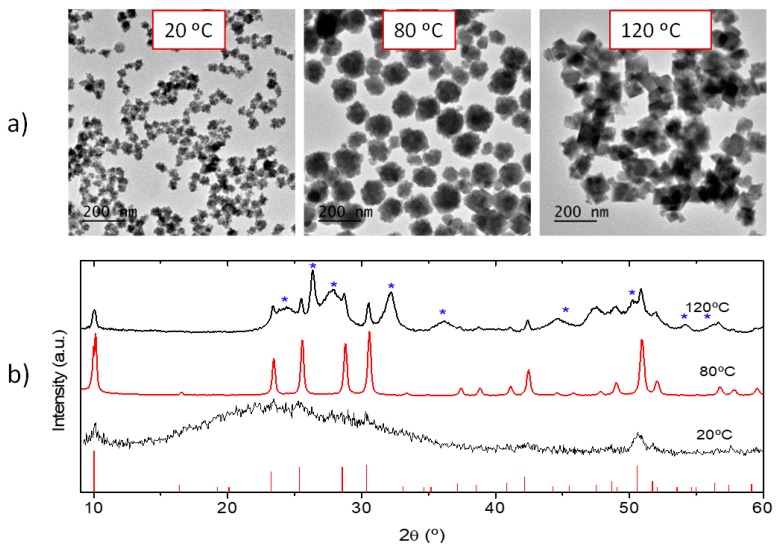
(**a**) TEM micrographs of nanoparticles obtained in the same conditions as described in Figure 1 except for the temperature, which was changed to the values shown in the labels. (**b**) Corresponding XRD patterns; ticks at the bottom correspond to cubic (H_3_O)Lu_3_F_10_ (PDF 04-016-7065) while asterisks on the top plot correspond to orthorhombic LuF_3_ (PDF 00-032-0612).

The reaction temperature (50 °C) was in good agreement with the temperature stability range (30–100 °C) reported for this phase [22]. In fact, when the synthesis was carried out at 20 °C, keeping the rest of experimental conditions constant, uniform nanoparticles were also obtained (Figure 2a) but they were mainly formed by an amorphous phase, as observed in the XRD pattern of Figure 2, bottom. Likewise, when the temperature was raised at 120 °C, highly aggregated, cube-like particles with heterogeneous size were observed (Figure 2a), which crystallized as a mixture of cubic (H_3_O)Lu_3_F_10_ and orthorhombic LuF_3_ (Figure 2b). However, when the reaction was conducted at a temperature of 80 °C, which is inside the temperature stability range reported for the cubic (H_3_O)Lu_3_F_10_ phase, such phase was the only one observed (Figure 2b) although the particles showed heterogeneous shapes and sizes between 50 and 150 nm (Figure 2a). In summary, the reaction temperature influences not only the crystalline phase obtained but also the particles size, shape, and the degree of dispersion, so that 50 °C was the lower temperature at which uniform particles of nanometric size, crystallizing in the (H_3_O)Lu_3_F_10_ phase, were obtained.

**Figure 3:**
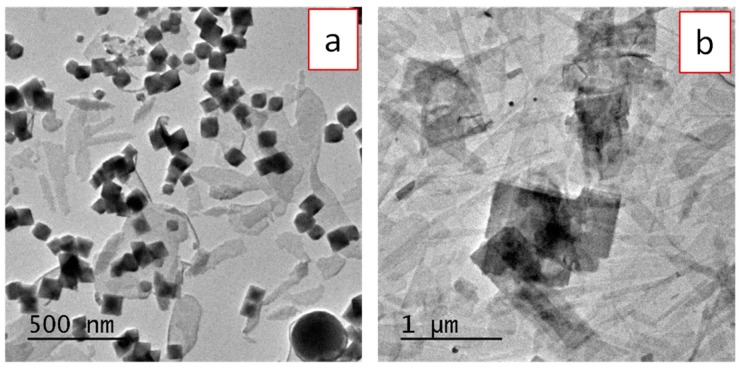
TEM micrographs of precipitates obtained after treating, at 50 °C for 15 h, EG/H_2_O solutions containing Lu(NO_3_)_3_·6H_2_O (0.2 M) and NaBF_4_ (0.18 M) at different EG/H_2_O ratios: 50/50 (**a**) and 0/100 (**b**).

Not only the temperature was an important experimental parameter to obtain uniform, nanometer size particles crystallizing as (H_3_O)Lu_3_F_10_ phase, but other experimental conditions also affected the nature of the precipitate. Thus, when no water was added to the solution, no precipitate was detected, which indicated that the hydration water of Lu(NO_3_)_3_·6H_2_O was not enough as to produce an efficient [BF_4_]^−^ hydrolysis. Water contents above 10% v/v were, however, not appropriate to get homogeneous precipitation, as demonstrated by the TEM micrographs of Figure 3, which correspond to the heterogeneous precipitates obtained using 50% (Figure 3a) and 100% (Figure 3b) water content.

**Figure 4:**
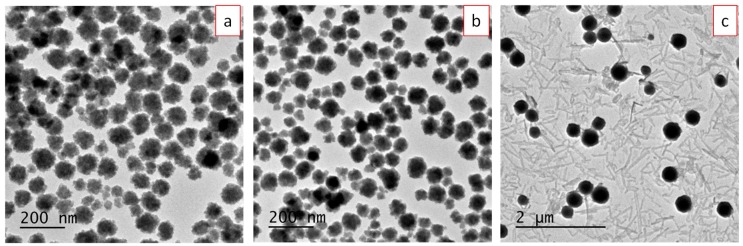
TEM micrographs of precipitates observed after treating, at 50 °C for 15 h, EG/H_2_O (90/10) solutions containing NaBF_4_ (0.18 M) and different Lu(NO_3_)_3_·6H_2_O contents: 0.05 M (**a**), 0.1 M (**b**), and 0.5 M (**c**).

Finally, the Lu(NO_3_)_3_·6H_2_O concentration was also a key parameter to obtain uniform particles of nanometric size. Thus, decreasing concentration to 0.1 and 0.05 M led to the precipitation of rounded particles with broad size distribution while increasing it to 0.5 M yielded a heterogeneous precipitate consisting of a mixture of spheres and needle-like particles (Figure 4).

**Figure 5:**
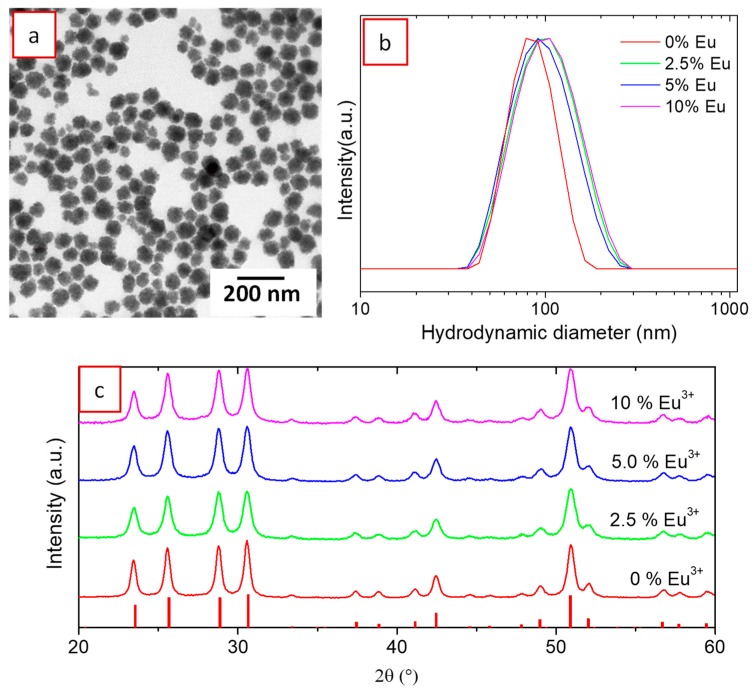
(**a**) TEM micrograph of 10% Eu^3+^ doped (H_3_O)Lu_3_F_10_ nanoparticles; (**b**) DLS curve of (H_3_O)Lu_3_F_10_ nanoparticles undoped and doped with 2.5%, 5%, and 10 mol% Eu^3+^; (**c**) corresponding XRD patterns. Ticks at the bottom of (c) correspond to cubic (H_3_O)Lu_3_F_10_ (PDF 04-016-7065).

In summary, the optimum conditions to obtain uniform, well-dispersed nanoparticles crystallizing as (H_3_O)Lu_3_F_10_ as the unique phase were those shown in Figure 1. Using such conditions and adding stoichiometric amounts of Eu(NO_3_)_3_·6H_2_O to the starting solutions, we proceeded to the synthesis of (H_3_O)Lu_3_F_10_ nanoparticles doped with different Eu^3+^ contents (Eu/(Eu + Lu) molar ratios = 2.5%, 5.0%, and 10%) in order to optimize the luminesce of the nanophosphors. Doping with Eu^3+^ did not produce significant modification of the nanoparticles morphology, as observed in the TEM image of Figure 5, which shows the 10% Eu-doped nanoparticles, the highest doping content used in this study. Likewise, the mean hydrodynamic size was around 90 nm, although the particle size distribution was slightly broader for the doped nanoparticles (Figure 5b). The doped nanoparticles showed very similar XRD patterns to the undoped ones (Figure 5c), indicating crystallization in the (H_3_O)Lu_3_F_10_ phase, although a slight shift of the reflections toward lower 2θ values could be observed after a careful examination of the patterns. Unit cell parameters were calculated from the XRD patterns of the undoped and doped materials using the Rietveld method. The unit cell volume obtained for the 10% Eu-doped sample was 3543.70 Å^3^, to be compared to 3515.25 Å^3^ corresponding to the undoped material. The observed volume expansion is consistent with Eu^3+^ incorporation due to the higher ionic radius of Eu^3+^ (1.066 Å) compared with that of Lu^3+^ (0.977 Å).

### 3.2. Luminescent Properties

**Figure 6:**
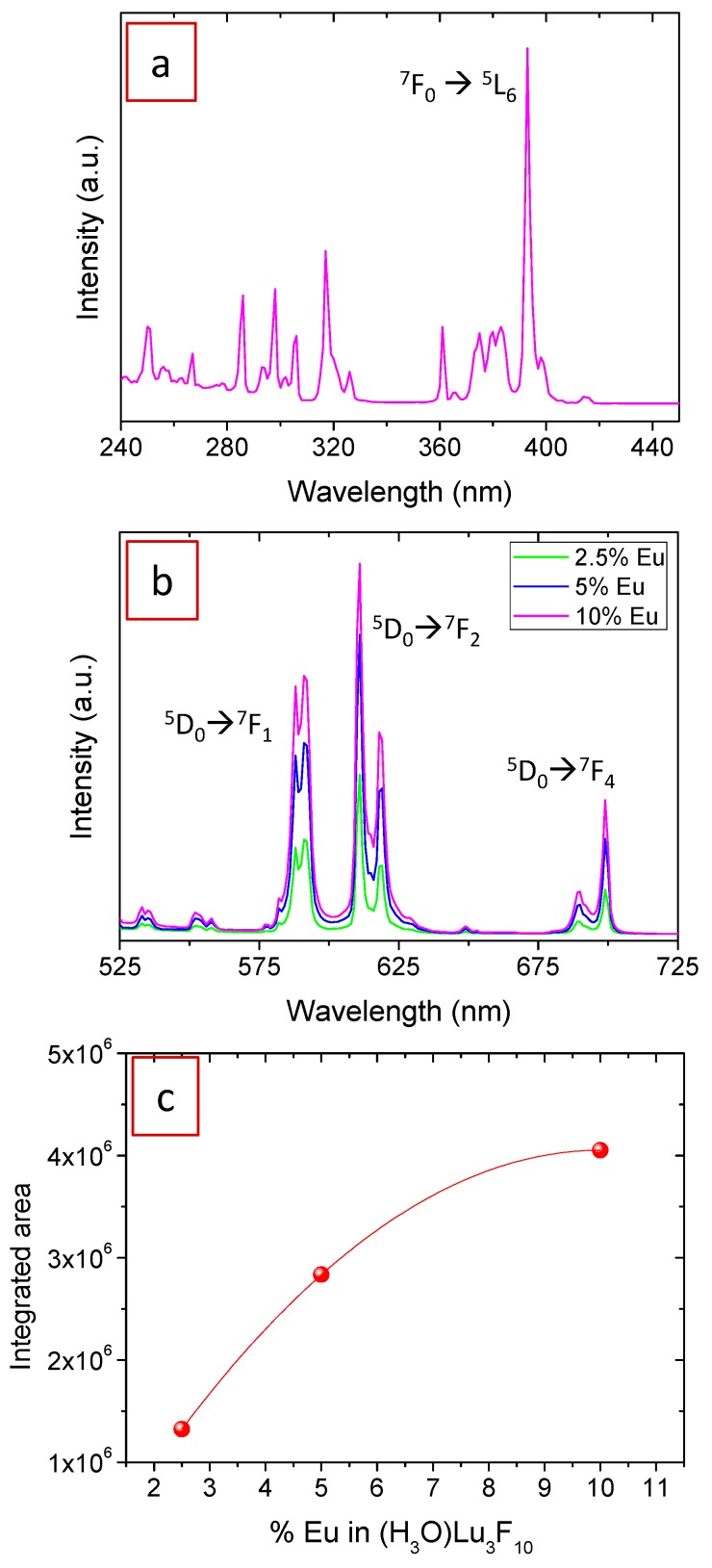
(**a**) Excitation spectrum of 10% Eu^3+^-doped (H_3_O)Lu_3_F_10_ nanoparticles recorded at an emission wavelength of 611 nm; (**b**) emission spectra of (H_3_O)Lu_3_F_10_ nanoparticles doped with 2.5%, 5%, and 10% Eu^3+^ recorded under an excitation wavelength of 394 nm; (**c**) integrated area, from 525 to 725 nm, of the spectra shown in (b) versus Eu content.

The excitation spectrum of the Eu^3+^-doped samples were recorded at an emission wavelength of 611 nm because at this value the most intense emission band was observed. The spectra of the three samples are very similar to each other and the one corresponding to the 10%Eu^3+^-containing sample is shown in Figure 6a as an example. It shows a set of narrow, well-defined bands in the UV region, which correspond to the f–f electronic transitions in Eu^3+^ ions. The most intense band corresponds to the ^7^F_0_ → ^5^L_6_ transition and it is located at 394 nm, in good agreement with the literature on Eu^3+^-doped fluorides [12,27]. Figure 6b shows the emission spectra of the Eu^3+^-doped nanoparticles while exciting the samples at 394 nm. They show the characteristic bands corresponding to the transitions from the ^5^D_0_ level to the ^7^F_J_ manifold, as indexed in the figure. The most intense band in all samples was observed at 611 nm and it corresponds to the forced electric dipole transition (^5^D_0_ → ^7^F_2_), which is sensitive to the site symmetry of the Eu^3+^ ions. The higher intensity of this band compared to the one corresponding to the magnetic dipole transition at 591 nm (^5^D_0_ → ^7^F_1_) indicates that Eu^3+^ ions are located in a non-centrosymmetric site. This result is in agreement with the D_2h_ site symmetry of Lu in the Fd-3m structure of (H_3_O)Lu_3_F_10_, where Eu is located in the doped material [22]. This is a favorable feature of our Eu^3+^:(H_3_O)Lu_3_F_10_ nanoparticles as the higher the intensity ratio between the electric and magnetic dipole transitions, the purer the red emission is. A similar intensity ratio between both emission bands was observed for Eu^3+^:NaLuF_4_ nanowires [28] while Eu^3+^ doped into other lutetium-based fluoride matrices, such as BaLuF_5_ [29,30] and LuF_3_ [12] showed a higher intensity for the magnetic dipole transition. 

It can be observed from Figure 6b that the intensity of the emission bands increased with increasing Eu^3+^ doping level because of the increased number of emission centers (Eu^3+^ ions). The value corresponding to the integrated area of the emission spectra has been plotted versus Eu content in Figure 6c. It can be observed that the emission does not increase linearly with increasing Eu content, which suggests the existence of a concentration quenching effect. Such an effect is the consequence of the emitting centers being so close together as to allow energy transfer to one another. The transfer process increases the probability that the photons travelling through the crystal may encounter luminescence killers (such as impurities or grain limits) eventually decreasing the intensity of the luminescence. 

**Figure 7:**
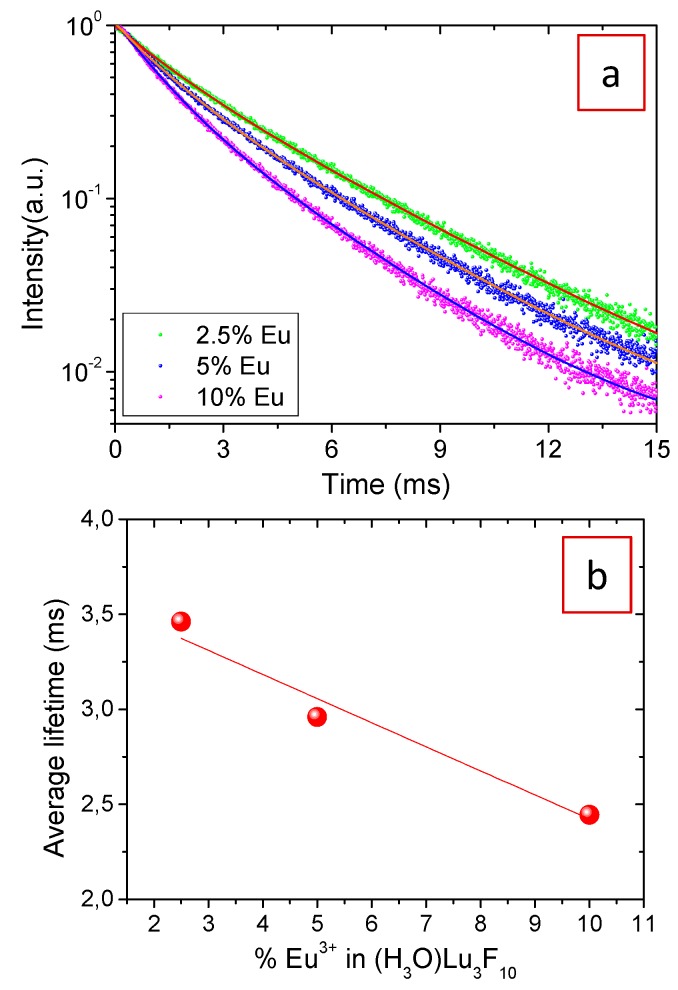
(**a**) Experimental (dots) and fitted (solid lines) temporal decays for the ^5^D_0_ → ^7^F_2_ transition (611 nm) of the Eu^3+^:(H_3_O)Lu_3_F_10_ nanoparticles with different Eu^3+^ contents; (**b**) average lifetime values calculated from such decays.

To confirm the presence of concentration quenching, the decay of the emission originated in the ^5^D_0_ → ^7^F_2_ transition (at 611 nm) was recorded for all three Eu^3+^-doped samples. Figure 7a shows the experimental decay curves as well as the fitted curves corresponding to a double exponential decay function of the form:

*I*(*t*) = *I*_01_ exp(−*t*/*τ*_1_) + *I*_02_ exp(−*t*/*τ*_2_) (1)

where *I*(*t*) is the luminescence intensity, *t* is the time after excitation, and *τ_i_* (*i* = 1, 2) is the decay time of the *i*-component, with initial intensity *I*_0i_. The fitting parameters are summarized in Table 1, along with the average lifetime values, <*τ*>, defined as:

(2) $$\langle \tau \rangle =\frac{\int_{t_{0}}^{t_{f}}tI\left(t\right)dt}{\int_{t_{0}}^{t_{f}}I\left(t\right)dt}=\left(\tau_{1}^{2}I_{1}+\tau_{2}^{2}I_{2}\right)/\left(\tau_{1}^{}I_{1}+\tau_{2}^{}I_{2}\right)$$

where *t_f_* represents a final time where the luminescence signal reaches the background, while *t*_0_ represents a time delay after the excitation pulse from where the luminescence decay is analyzed. 

The existence of two different decay times has been assigned in the related literature to the fact that the Eu^3+^ ions located at the nanoparticle surface, such as OH-species, are affected by surface defects that are known to be luminescence quenchers, thus increasing the electrons decay rate. On the contrary, such quenchers do not affect Eu^3+^ ions inside the particle and they, therefore, show a slower decay [31]. The size of our particles is relatively large (tens of nanometers) and, consequently, the surface-to-volume ratio is small, i.e., surface-related effects are much weaker than the bulk ones. However, the peculiar zeolitic structure of the material, with cavities linked together by tunnels where water molecules are located, increases the number of Eu^3+^ ions affected by luminescence quenchers. This explains the important contribution of the short decay time to the average lifetime, as observed in Table 1. The average decay times, shown in Figure 7b, decrease with increasing Eu content, which indicates that a concentration quenching effect is taking place over the entire concentration interval analyzed. The most efficient doping level was therefore, 2.5 mol% Eu as it shows the highest lifetime value. However, from a practical point of view, the most interesting nanoparticles for possible use as luminescent bioprobes were those doped with 10 mol% Eu^3+^ which present the highest emission intensity because the number of emitting centers compensates the concentration quenching effect. The 10% Eu-doped sample was therefore subsequently used for the evaluation of the X-ray attenuation properties.

### 3.3. X-ray Attenuation Properties

**Figure 8:**
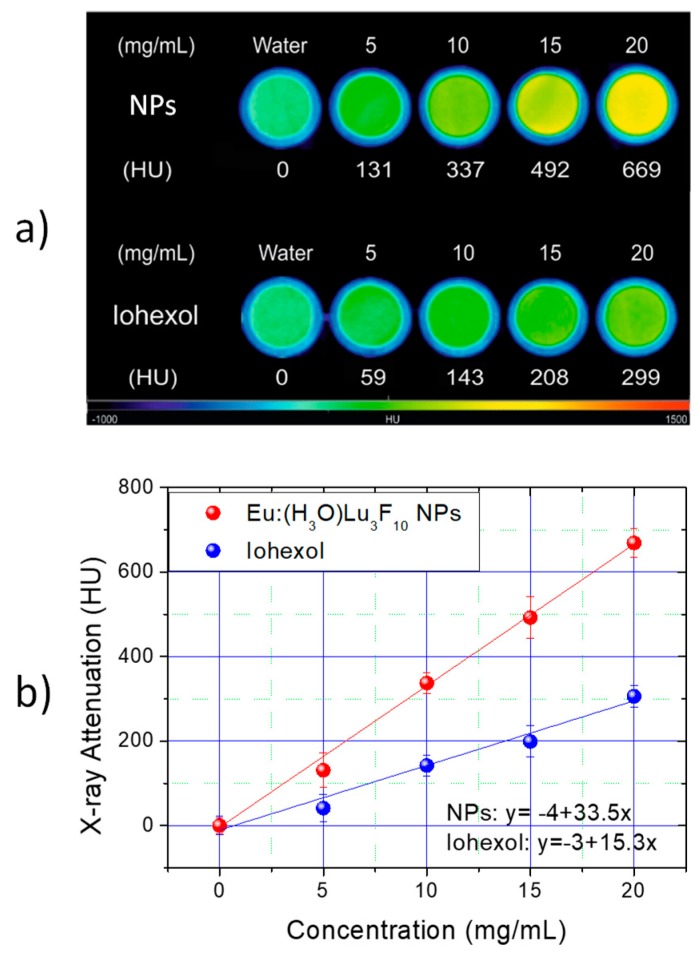
Computed tomography (CT) phantom images (**a**) and HU values (**b**) of 10% Eu-containing (H_3_O)Lu_3_F_10_ nanoparticles and Iohexol at different concentrations in water.

To evaluate the X-ray attenuation capacity of the 10% Eu^3+^-doped (H_3_O)Lu_3_F_10_ nanoparticles, aqueous suspensions with different contents of the nanoparticles were prepared. Likewise, solutions containing the same concentration of a commercial, currently used CT CA (Iohexol) were also prepared for comparative purposes. The CT phantom images obtained from the wells containing each suspension are presented in Figure 8a. A change in the color of the phantoms was observed as the concentration of the nanoparticles increased, unlike the Iohexol solutions, whose color hardly changed with concentration. The color change is proportional to the X-ray attenuation produced by the different suspensions, which suggests that the attenuation capacity was greater for the Lu-based nanoparticles than for Iohexol. The attenuation values in Hounsfield units were subsequently obtained from the phantom images and are shown in Figure 8b. The attenuation values increased linearly with the concentration for both Lu-based nanoparticles and Iohexol. The slope of the line, which is directly proportional to the X-ray attenuation capacity of the CA, is significantly higher for the nanoparticles than that for Iohexol. It can be concluded, therefore, that the Eu:(H_3_O)Lu_3_F_10_ nanoparticles are suitable as CT CAs and produce a much higher contrast than Iohexol. In addition, it is well known that in order to obtain sufficient contrast in an image obtained by CT, the local change in X-ray attenuation produced by the CA must be at least 100 HU [32]. To achieve this contrast, it was necessary to use local Iohexol concentrations of about 8 mg·cm^−3^, as shown in Figure 8b, while the same contrast could be achieved with less than 3 mg·cm^−3^ in the case of the Lu-based nanoparticles. This significant reduction in the dose of CA represents an important benefit for the patient.

## 4. Conclusions

We have demonstrated that uniform (H_3_O)Lu_3_F_10_ nanoparticles can be synthesized using a simple polyol-based method consisting of treating, at 50 °C for 15 h, an EG/H_2_O (90/10) solution containing Lu(NO_3_)_3_·6H_2_O (0.2 M) and NaBF_4_ (0.18 M). The reaction renders water-dispersible nanoparticles with a diameter of 55 nm that can be doped with different Eu^3+^ contents (2.5–10 mol%). From the analysis of the luminescent properties of the doped nanoparticles, it can be concluded that, although the 2.5% Eu-doped nanoparticles showed the highest lifetime value, those doped with 10% Eu^3+^ produced the highest emission intensity and are, therefore, the optimum candidates to be used for luminescence purposes. The X-ray attenuation of the latter nanoparticles was found to be much higher than that of a commercial CT CA, so that an attenuation of 100 HU could be obtained with an aqueous suspension containing a nanoparticle concentration of 3 mg/mL versus 8 mg/mL needed to produce the same attenuation with an aqueous solution of the commercial agent. These features make the Eu^3+^:(H_3_O)Lu_3_F_10_) nanoparticles good potential candidates to be used as bimodal bioprobes for luminescence bioimaging and X-ray computed tomography. This work represents an advance in the field of nanoparticle synthesis as it reports the fabrication of nanoparticles of a compound that had only been fabricated before in the form of a polycrystalline powder [22]. Future work is needed to assess the biocompatibility of the synthesized nanoparticles.

## Figures and Tables

**Table 1:** Lifetime values for the Eu^3+^:(H_3_O)Lu_3_F_10_ nanoparticles having different Eu^3+^ contents for the ^5^D_0_ → ^7^F_2_ transition (611 nm) after pulsed excitation at 394 nm.

% Eu^3+^	*τ*_1_ (ms)	*I*_1_ (%)	*τ*_2_ (ms)	*I*_2_ (%)	<*τ*> (ms)
2.5	3.90	63	1.54	37	3.46
5	3.47	55	1.30	45	2.96
10	3.08	43	1.15	57	2.44
